# An Integrated Analysis to Understand the Dysregulation of Innate Immune Response in Mouse Models of MASLD

**DOI:** 10.3390/cells15141298

**Published:** 2026-07-21

**Authors:** Yamini Goswami, Jyoti Gautam, Akash Baghel, Deepika Kumari, Phulwanti Kumari Sharma, M.R. Kamala Priya, Ruby Bansal, Farah Gulzar, Rajni Yadav, Guruprasad P. Aithal, Shalimar, Madhu Dikshit, Ruchi Tandon

**Affiliations:** 1 BRIC-Translational Health Science and Technology Institute, NCR Biotech Science Cluster, 3rd Milestone, Faridabad-Gurugram Expressway, Faridabad 121001, India; 2 All India Institute of Medical Sciences, New Delhi 110029, India; 3 National Institute for Health Research (NIHR) Nottingham Digestive Diseases Biomedical Research Centre, Nottingham University Hospital NHS Trust and University of Nottingham, Nottingham NG7 2UH, UK; 4 CSIR-Central Drug Research Institute, Lucknow 226031, India

**Keywords:** inflammasomes, NLRP3, AIM2, NLRC4, MASLD

## Abstract

**Highlights:**

Key Findings

- Canonical inflammasomes represent a shared innate immune signature across mouse models of MASLD, patients, and in vitro cell-based assays.
- A diet deficient in methionine-choline can lead to more pronounced activation of the innate immune response than the obesogenic diet.

Key Implications

- Choline availability modulates inflammasome-associated inflammatory responses under metabolic stress.
- Choline supplementation may represent a nutritional strategy to mitigate inflammation in MASLD.

**Abstract:**

Background: Dysregulation of innate immune pathways is an important contributor to the progression of metabolic dysfunction-associated steatotic liver disease (MASLD). Inflammasomes serve as key regulators of innate immunity. Emerging evidence suggests that nutrient status, particularly choline, may play an important role in inflammasome activation. However, there is no systematic study across different in vitro and in vivo experiments to demonstrate the same. Methods: Hepatic expression of canonical inflammasomes such as NLRP3, NLRC4, and AIM2 and downstream inflammatory cytokines was analyzed in the high-fat-high-fructose (HF-HF) and methionine-choline-deficient (MCD) diet-induced mouse models of MASLD, along with liver biopsies from MASLD patients (*n* = 20). Moreover, in vitro experiments were performed to analyze the potential of choline to modulate inflammasome activation under obesogenic conditions. Results: Our data suggest increased hepatic expression of IL-1β and IL-18, at both mRNA and protein levels, in both models. While MCD-fed mice showed elevated hepatic mRNA expression of all three inflammasomes, the HF-HF diet induced a selective upregulation of NLRP3. Consistently, MCD-fed mice showed stronger induction of IL-1β and IL-18. MASLD patients also demonstrated increased hepatic expression of inflammasomes and both cytokines. In vitro, choline supplementation attenuated free fatty acid- and fructose-induced cytokine elevation. Conclusions: Our data suggest the differential activation of canonical inflammasomes across experimental MASLD models. We also observed that choline availability can serve as a potential modulator of metabolic stress-induced inflammation, highlighting the importance of nutrient status in metabolic liver disease.

## 1. Introduction

Metabolic dysfunction-associated steatotic liver disease (MASLD) is a chronic, progressive condition affecting approximately 30–38% of the global adult population according to recent epidemiological estimates (2022–2024) [1]. Excess lipid accumulation in hepatocytes induces lipotoxicity and cellular stress, which activate innate immune pathways and recruit macrophages and neutrophils, the primary drivers of hepatic inflammation. This further triggers the activation of hepatic stellate cells and facilitates the progression of disease to the metabolic dysfunction-associated steatohepatitis (MASH). Since the innate immune activation is a hallmark of the transition of disease from MASLD to MASH, our study aimed to identify specific mediators of the innate immune system that could help in the detection of disease at an early stage.

**Scheme 1:**
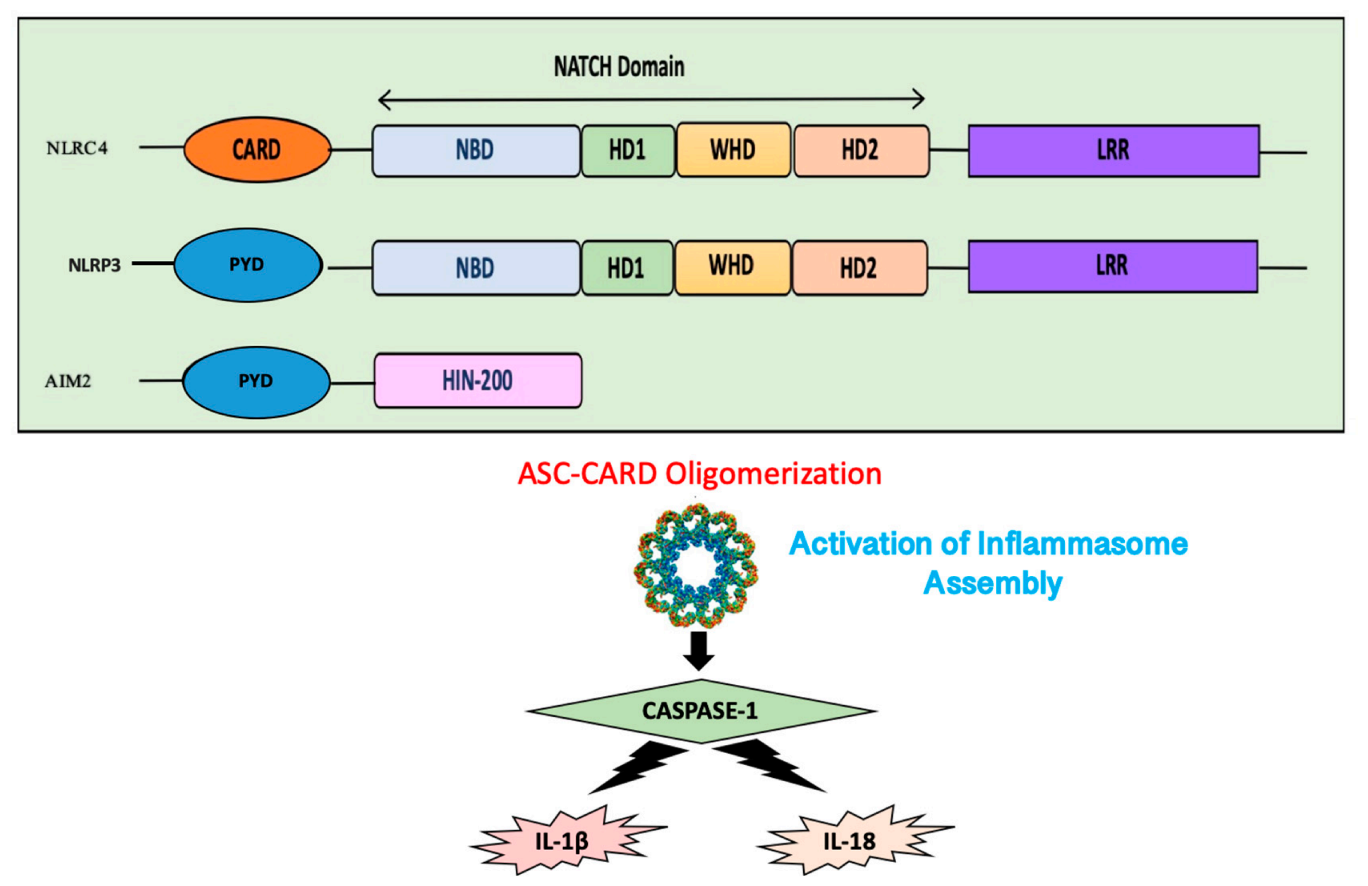
Domain structure of canonical inflammasomes NLRP3, NLRC4, and AIM2.

Inflammasomes are essential components of the innate immune response. Several members of the inflammasome family have been previously reported to play a central role in the development and progression of MASLD [2]. They are intracellular protein complexes activated by pathogen-associated molecular patterns (PAMPs) and danger-associated molecular patterns (DAMPs) in response to cellular insults, triggering inflammation [3]. The canonical inflammasome members in the current context induce the potent inflammatory response through the activation of Caspase-1 and subsequent release of interleukin-1β (IL-1β) and interleukin-18 (IL-18) [4]. In our current study, we undertook three canonical inflammasomes: (1) nucleotide-binding oligomerization domain (NOD), leucine-rich repeat (LRR) (NLRs) PYRIN domain (PYD) containing protein 3 (NLRP3), (2) NLR family CARD domain-containing protein 4 (NLRC4), and (3) Absent in melanoma 2 (AIM2). NLRP3 and NLRC4 are NLRs [5] with distinct PYRIN and CARD domains at their N-terminus, respectively [6], while AIM2 consists of an N-terminal PYD (1–87) and a C-terminal HIN domain (138–343) [7]. Domain structures of all three canonical inflammasomes—NLRP3, NLRC4, and AIM2—are given in Scheme 1.

Multiple studies implicate the role of NLRP3 in MASLD pathogenesis, including its aberrant activation in liver tissues of patients and diet-induced mouse models, protection from steatohepatitis in NLRP3 knockout mice, elevated NLRP3 levels in CDAA-fed C57BL/6 mice, and its excessive activation in metabolic disorders linked to hepatic insulin resistance and weight gain [8,9,10]. The roles of NLRC4 and AIM2 in the progression of MASLD, however, remain unclear in mice fed with different diets. There is some ambiguity with regard to their expression levels in mice fed a high-fat diet [11]. In another study, mice fed a methionine- and choline-deficient diet (MCD) showed increased expression of NLRP3 and AIM2, although the involvement of NLRC4 in this model was not reported [12].

To address the existing knowledge gaps, we designed a comprehensive study integrating two diet-induced murine models of MASLD. In the first study, mice were fed a high-fat, high-fructose (HF-HF) diet to replicate obesity-related metabolic stress, and in another study, mice were fed a methionine-choline-deficient (MCD) diet to induce liver injury and fibrosis without obesity. A detailed biochemical, metabolic and histopathological analysis was done in both the study groups. Expression levels of inflammatory markers such as IL-1β, IL-18, and associated inflammasome components were also analyzed in the liver samples in both studies using mice, along with the liver biopsy samples from MASLD patients. Moreover, in vitro experiments were also done using HepG2 cells and differentiated THP-1 cells to reconfirm the above observations under defined experimental conditions.

## 2. Materials and Methods

### 2.1. Animals

Male C57BL/6 mice (8–12 weeks old, weighing 20–25 g) were received from the Small Animal Facility of the Translational Health Science and Technology Institute (THSTI), Faridabad, and sheltered in individual cages with ventilation as per the institutional guidelines for working with animals. All the protocols were approved by the Institutional Animal Ethics Committee (IAEC) of THSTI, Faridabad, India (Protocol approval nos. IAEC/THSTI/163 and 168).

Animals were housed individually in ventilated cages at the Small Animal Facility, BRIC-Translational Health Science and Technology Institute (THSTI), Faridabad, India. Standard environmental conditions were maintained, including a 12 h light/12 h dark cycle, temperature of 25 ± 2 °C, and relative humidity of 60 ± 10%. Environmental enrichment was given by providing bedding material and nesting support that allowed natural behaviors and reduced stress.

Mice had ad libitum access to food and water throughout the study. Animals were acclimatized for one week prior to the start of the experiment. Routine husbandry practices, including cage cleaning and health monitoring, were carried out according to institutional guidelines.

### 2.2. Establishment of Mouse Models of MASLD Using Different Diets

**Scheme 2:**
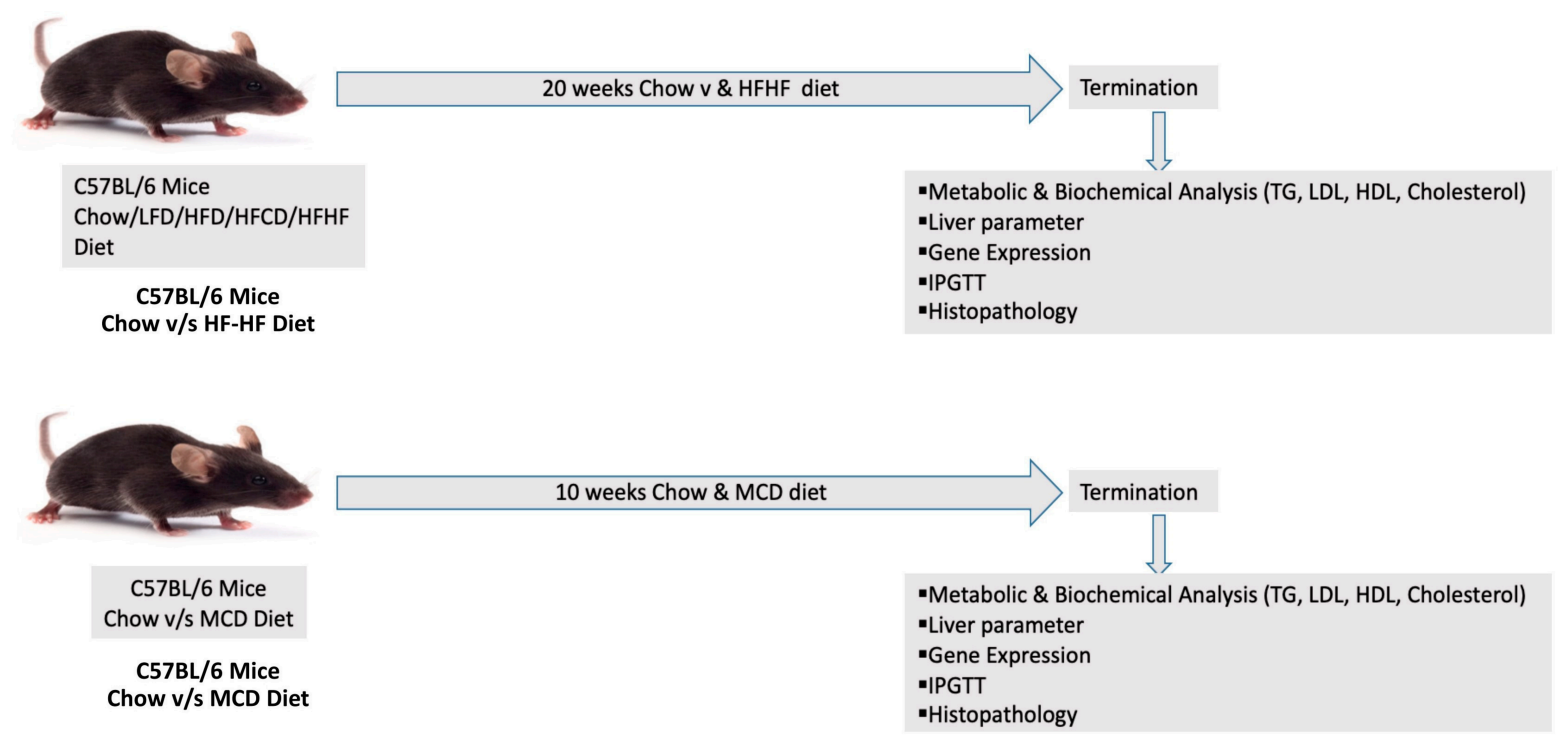
The development of a mouse model of MASLD using C57BL/6 mice, a 20-week study using HF-HF diet (**upper panel**) and MCD diet for 10 weeks (**lower panel**).

Two dietary groups of C57BL/6J mice were used in this study: mice fed with control chow diet (1324P, Altromin international, Lage, Germany) and HF-HF diet (D16030909 (Research Diet Inc., New Brunswick, NJ, USA)), that were maintained for 20 weeks, and a separate chow control and MCD diet (A02082002 BR, Research Diet Inc., New Brunswick, NJ, USA)-fed group of mice for 10 weeks as shown in Scheme 2. Each group had 6 mice (*n* = 6). Intraperitoneal glucose tolerance test (IPGTT) was performed. Body weight, blood glucose, lipid profiles, and body mass index (BMI) were recorded. At the end of the experimental period, mice were euthanized, and blood and liver tissues were collected, washed, and stored at −80 °C for further analysis. All the diets used in this study were obtained from Research Diet Inc., New Brunswick, NJ, USA. Detailed compositions of the diets are given in Supplementary Table S1.

(BMI = Ratio between body weight and the square of the body length from nose to anus (g/cm^2^)).

### 2.3. Biochemical Analysis

Blood was collected in EDTA-coated (1.8 mg/mL) microcentrifuge tubes after 6 h of fasting and centrifuged at 1500× *g* for 15 min at 4 °C to obtain plasma. The supernatant was collected and stored at −80 °C until used for the following studies using the kits from Randox. Plasma triglycerides (Cat. No. TR2774, LOT No. 220218001), cholesterol (Cat. No. CH2773, LOT No. 211209001), low-density lipoprotein (Cat. No. CH2776, LOT No. 220106001), and high-density lipoprotein (Cat. No. CH2775, LOT No. 220106002) were measured in plasma samples using the manufacturer’s protocols and absorbance was recorded using a Spark^®^ Multimode Microplate Reader (Model SPARK 10M, REF 30086375; manufactured by Tecan Group Ltd., Männedorf, Switzerland).

### 2.4. IPGTT and HOMA-IR Analysis

Animals were fasted for 6 h for the assessment of the intraperitoneal glucose tolerance test (IPGTT). Two grams of d-Glucose/kg body weight was injected intraperitoneally (IP) into the fasted mice, and blood glucose concentrations were measured at 0, 15, 30, 60, 90, and 120 min after administration of glucose, using a glucometer (Accu-Chek, Roche, Basal, Switzerland REF 07444141200). The area under the curve (AUC) was calculated as described earlier [13]. The homeostasis model assessment of insulin resistance (HOMA-IR) was calculated using the formula: fasting insulin (μIU/mL) × fasting glucose (mg/dL)/405 as previously described [14].

### 2.5. Histological Analysis

For histopathological evaluation, liver tissues from mice were dissected and fixed in formalin. Paraffin-embedded blocks were used to cut sections of liver and were stained with hematoxylin and eosin (H&E) and Masson’s trichrome (MT) stains for evaluation of various histopathological features for grading and staging using the MASH Clinical Research Network (CRN) grading and staging system. Histological examinations, imaging, and blinded scoring were conducted at the All India Institute of Medical Sciences, New Delhi, India. Histological examination and representative images of H&E-stained liver sections were captured using an Olympus CX43RF T2 light microscope (Olympus Corporation, Tokyo, Japan).

### 2.6. Needle Biopsy of MASLD Patients and Histopathological Analysis

Liver biopsy was performed by a treating physician or an interventional radiologist following an ultrasound-guided percutaneous plugged approach. The biopsy samples were fixed using 10% neutral formalin, processed, and embedded in paraffin. Sections of 4-micron thickness were stained with H&E (hematoxylin and eosin) along with MT (Masson’s trichrome) staining and evaluated by an expert pathologist. Samples with at least 11 portal areas were considered adequate for further processing. The MASH-CRN scoring pattern was used to analyze the stage of fibrosis, and a MASH activity score > 4 points was used to consider MASH. Fibrosis was graded from F0 to F4, where F0 represents the absence of fibrosis, F1 represents periportal or perisinusoidal fibrosis, F2 represents perisinusoidal and portal/periportal fibrosis, F3 represents bridging fibrosis, and F4 represents cirrhosis. Fibro scan data of individual patients (*n* = 20) are given in Supplementary Table S2. All the protocols to obtain biopsy liver tissues from MASLD patients were approved by the Institute Ethics Committee, All India Institute of Medical Sciences, New Delhi, India. (Reference Number: IEC-223/09.04.2021, OP-02/04.11.2022).

### 2.7. In Vitro Assays

#### 2.7.1. Cell Culture

THP-1 human monocytic cells (ATCC TIB-202) and HepG2 human hepatocellular carcinoma cells (ATCC HB-8065) were obtained from the American Type Culture Collection (ATCC, Manassas, VA, USA). THP-1 cells were maintained in RPMI-1640 medium (Gibco, Thermofisher scientific, Waltham, MA, USA), while HepG2 cells were cultured in Dulbecco’s Modified Eagle Medium (DMEM; Gibco, USA), supplemented with 10% fetal bovine serum (FBS) and 0.5% penicillin-streptomycin (100 U/mL penicillin and 100 μg/mL streptomycin). Cells were maintained at 37 °C in a humidified incubator with 5% CO_2_. HepG2 cells were passaged upon reaching approximately 70–80% confluence using 0.25% trypsin-EDTA, whereas THP-1 cells were maintained in suspension culture according to ATCC recommendations. All experiments were performed using cells between passages 5 and 10. Cells were routinely monitored for morphology and tested periodically to ensure the absence of mycoplasma contamination. Primary human hepatocytes from healthy donors were procured from Lonza Group Ltd. (Basel, Switzerland) and cultured according to the manufacturer’s instructions.

#### 2.7.2. Fatty Acid Induced IL-1β Induction Analysis in HepG2 Cells

HepG2 cells were seeded at a density of 8 × 10^3^ cells/well in a 96-well plate and allowed to attach overnight at 37 °C in a humidified incubator with 5% CO_2_. Cells were then incubated for an additional 16 h at 37 °C with a mixture of free fatty acids (FFA) consisting of 333.3 µM oleic acid (OA) and 166.7 µM palmitic acid (PA) (2:1 molar ratio) in 10% BSA to give a final concentration of 500 µM FFA. Final concentration of BSA in the wells was 1% in both the treated and control groups. Cells were then washed, fixed with paraformaldehyde (4%), and stained with Nile Red (7.5 µg/mL) and DAPI (2 µg/mL) for 30 min. Fluorescence was measured (SpectraMax M2, Molecular Devices, Silicon Valley, CA, USA), and images were captured (In Cell Analyzer 6000, GE Healthcare, Chicago, IL, USA). Levels of IL-1β protein were quantified by ELISA. mRNA expression analysis was done by qRT-PCR using SYBR chemistry after Trizol-based RNA extraction and cDNA synthesis.

#### 2.7.3. Inflammasome Activation Analysis Using Differentiated THP-1 Cells

THP-1 cells were plated at a density of 2 × 10^4^ cells/well in a 96-well plate and differentiated with PMA (50 nM) for 48 h. After PBS washes, cells were maintained in RPMI medium containing 10% FBS and 0.5% pen-strep, then primed with LPS (100 ng/mL) for 2 h, followed by stimulation with ATP (2.5 mM) for an additional 2 h at 37 °C. IL-1β mRNA and protein levels were analyzed as described for HepG2 cells.

#### 2.7.4. Co-Culture Experiment to Check the Effect of Choline on Inflammation-Induced by Palmitic Acid and Fructose Treatment

One lakh THP-1 cells were treated with 50 nM phorbol 12-myristate 13-acetate(PMA) and allowed to differentiate for 48 h. Cells were then incubated in fresh RPMI-1640 medium for an additional 24 h. Immediately after differentiation, a direct co-culture was established by seeding HepG2 cells (5 × 10^5^) together with differentiated THP-1 cells at a 5:1 ratio (HepG2:THP-1) in the same culture well, thereby allowing direct cell–cell contact and exchange of soluble factors. The co-culture was pre-treated with choline bitartrate (100 µM or 300 µM) for 30 min, after which cells were exposed to palmitic acid (100 µM) and fructose (100 mM) for 48 h. Cells were then analyzed by qRT-PCR for mRNA expression analysis.

### 2.8. IL-1β and IL-18 Protein Expression Analysis by Western Blot

Liver tissues (25 mg) from MCD and HF-HF mice were lysed and analyzed for IL-1β and IL-18 protein by Western blot using Bio-Rad systems. Primary (1:1000) and secondary (1:3000) antibodies from Cell Signaling Technology, Danvers, MA, USA were used in PBS with 5% BSA. Detection was performed with ECL reagents (Thermo Fisher) followed by image analysis using the GelDoc (Bio-Rad, Hercules, CA, USA).

### 2.9. RNA Isolation and Quantification

RNA was extracted from mouse liver tissue (5–10 mg), primary human hepatocytes (Lonza Group Ltd., Basel, Switzerland) or HepG2 and THP-1 cells in either mono- or co-culture setups using TRI Reagent (Sigma-Aldrich, Burlington, MA, USA) and following the manufacturer’s instructions. The quality and concentration of RNA were analyzed using the NanoDrop (Thermo Scientific, Waltham, MA, USA).

### 2.10. Gene Expression Studies

cDNA was synthesized from RNA (2 µg) using the cDNA Kit (Applied Biosystems, Thermo Scientific, Waltham, MA, USA). qRT-PCR was performed on a QS6 system using the master mix containing SYBR Green dye (TaKaRa Bio, Kutasu, Shiga, Japan) and gene-specific primers (Table 1). Thermal cycling included 40 cycles of 95 °C for 3 s and 60 °C for 30 s. GAPDH or 18S was used as the housekeeping gene for normalization of gene expression.

### 2.11. Statistical Analysis

Data (*n* = 3) were analyzed using GraphPad Prism 8.3 with one- or two-way ANOVA followed by Bonferroni’s test. Normality of data distribution was analyzed using the Shapiro–Wilk test before performing ANOVA. Results are expressed as mean ± SD, with * *p* < 0.05 considered significant. Asterisks indicate comparisons between diet groups and the chow group.

## 3. Results

### 3.1. Experiments in Mice

#### 3.1.1. Mice Fed with Different Diets Show Differential Gene Expression Patterns of Nlrp3, Nlrc4, Aim2 and Their Downstream Signaling Partners in the Liver Tissues

**Figure 1:**
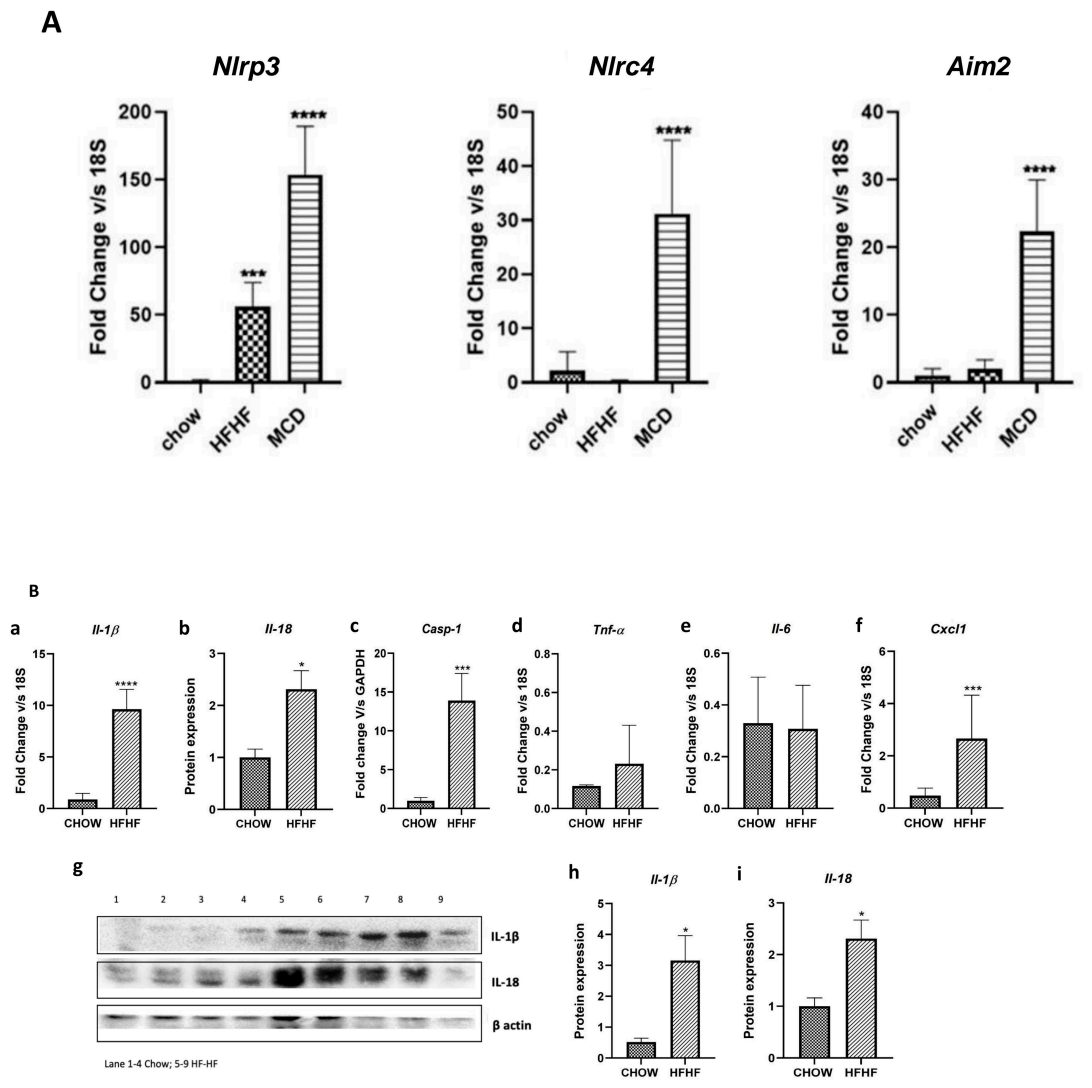

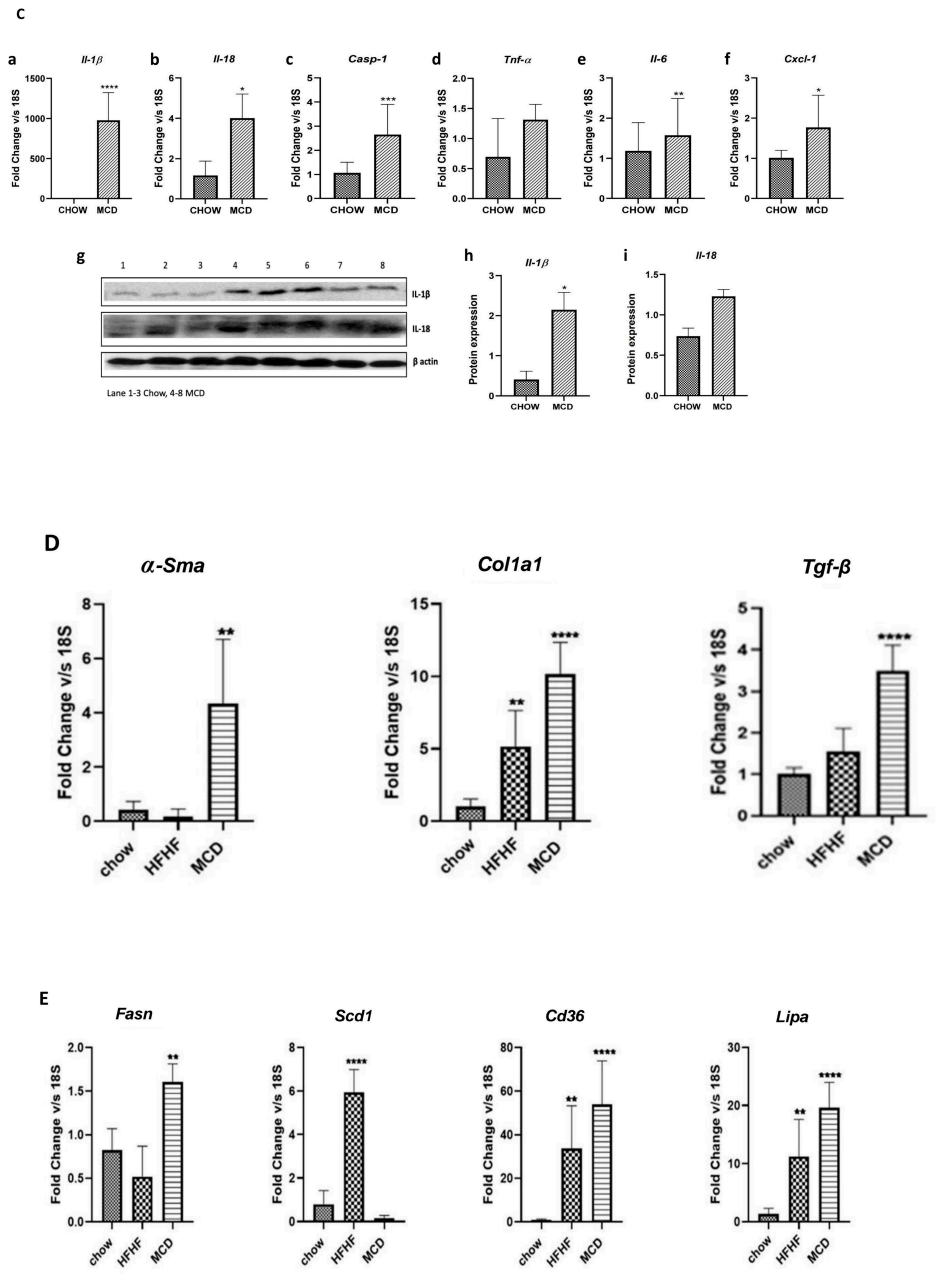
Expression of canonical inflammasomes in mouse models of MASLD; (**A**) mRNA expression levels of *Nlrp3*, *Nlrc4*, and *Aim2* in the liver tissues of C57BL/6 mice fed with MCD and HF-HF diets in comparison with mice fed with a chow diet; (**B**) mRNA expression studies of (**a**) *Il-1*β, (**b**) *Il-18*, (**c**) *Caspase-1*, (**d**) *Tnf-*α, (**e**) *Il-6*, (**f**) *Cxcl1* and protein expression of inflammatory cytokines (**g**) western blot image of *IL-1β, IL-18*, lane 1–4-Chow; lane 5–8-HF-HF(**h**) densitometric analysis of western blot (fold change vs. *β-actin*) of *Il-1β* (**i**) densitometric analysis of western blot image (fold change vs. *β-actin*) of *Il-18* in the liver tissues of C57BL/6 mice fed with the HF-HF diet, and (**C**) mRNA expression studies of (**a**) *Il-1β*, (**b**) *Il-18*, (**c**) *Caspase-1*, (**d**) *Tnf-*α, (**e**) *Il-6*, (**f**) *Cxcl1* and protein expression of inflammatory cytokines (**g**) Western blot image of *Il-1β*, *Il-18*, lane 1-3-Chow; lane 4-8-HF-HF (**h**) densitometric analysis of western blot image (fold change vs. β-actin) of *Il-1β* (**i**) densitometric analysis of western blot (fold change vs. β-actin) of *Il-18* in the liver tissues of C57BL/6 mice fed with the MCD diets; (**D**) mRNA expression levels of fibrotic markers; α*-Sma*, *Col1a1* and *Tgf*-*β* in the liver tissues of C57BL/6 mice fed with HF-HF and MCD diets; (**E**) mRNA expression levels of genes related to fat metabolism; *Scd-1*, *Fasn*, *Cd36*, and *Lipa* in the liver tissues of C57BL/6 mice fed with HF-HF and MCD diets in comparison with mice fed with a chow diet. Gene expression is expressed as fold change (2^−ΔΔCT^) relative to control and normalized to 18S. Data are represented as mean ± SEM, *n* = 6 per group, and statistical analysis consisted of one-way ANOVA or two-way ANOVA followed by Bonferroni’s test (* *p* < 0.05; ** *p* < 0.01; *** *p* < 0.001; **** *p* < 0.0001 for control vs. all other groups).

**Scheme 3:**
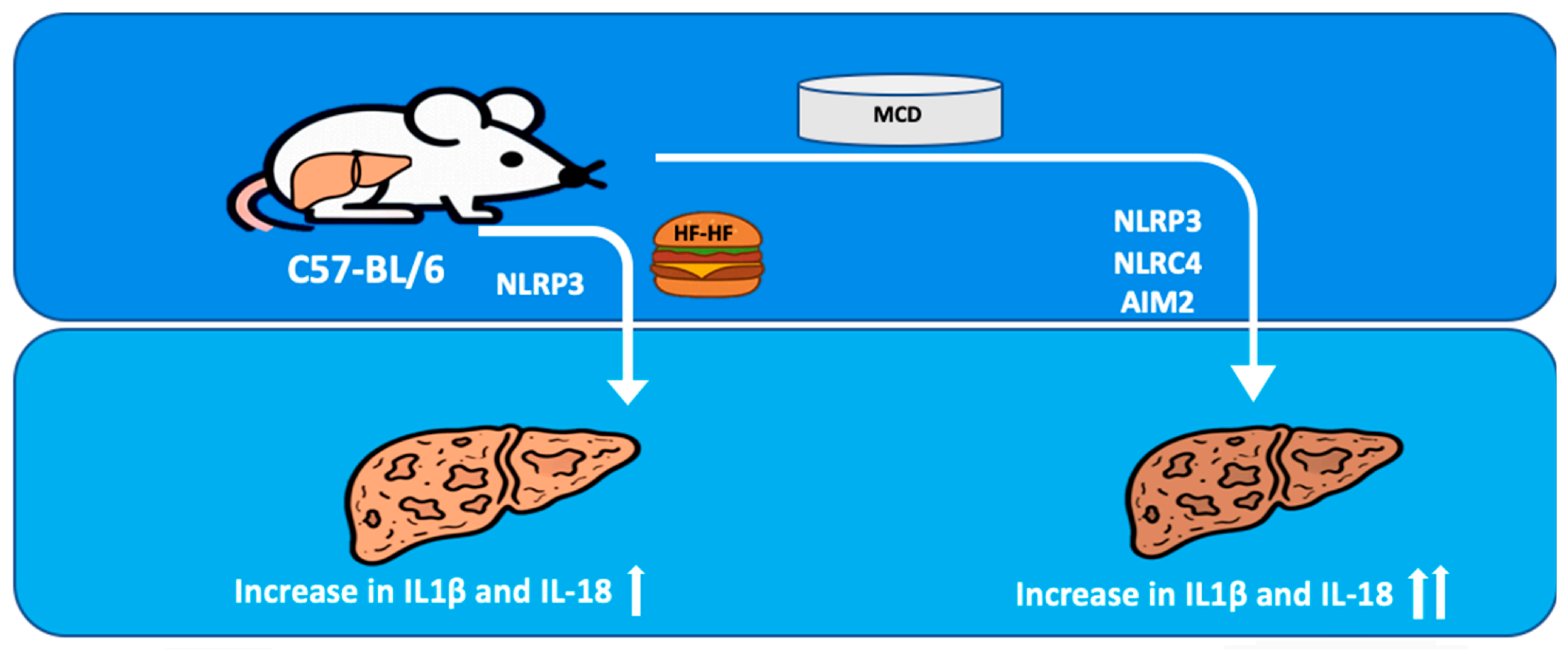
Differential expression of inflammasomes in HF-HF and MCD diet induced mouse models of MASLD.

Gene expression analysis of members of the inflammasome family was done using the liver tissues of C57BL/6 mice fed with HF-HF and MCD diets in comparison with mice fed with a chow diet by qRT-PCR analysis (Figure 1A). Our data showed high expression levels of all three inflammasomes; *Nlrp3*, *Nlrc4* and *Aim2* in the MCD group in comparison with the chow diet. Expression levels of *Nlrp3* mRNA were also high in mice fed with HF-HF diet compared to the chow diet; however, it was several folds lower than the MCD group. In contrast to the MCD group, no significant increase in the expression of *Nlrc4* or *Aim2* was observed in the HF-HF diet group. Expression levels of their downstream family members *Il-1β*, *Il-18* were also analyzed by gene (Figure 1B(a,b) and Figure 1C(a,b)) and protein expression analysis (Figure 1B(g–i) and Figure 1C(g–i), respectively) and were found to be higher in both the diet groups in accordance with the inflammasome expression profiles. All the data were normalized against the expression levels of *18s*, which were used as internal controls. Summary of these findings shown in Scheme 3.

#### 3.1.2. Mice Fed with Different Diets Show Differential mRNA Expression of Key Hallmarks of Steatosis and Fibrosis in the Liver Tissues

We aimed to study the relative modulation of the expression levels of genes associated with the key hallmarks of fibrosis and steatosis in the liver tissues of mice fed with both diets. Significantly high mRNA expression levels of *α-Sma*, *Col1a1*, and *Tgf-β* were observed in the liver tissue of MCD diet-fed mice [15], while the HF-HF group showed slightly increased levels of *Col1a1* compared to the chow-fed mice as reported previously [16,17] and correlated further with the histochemical analysis (Figure 1D, Supplementary Table S4).

Significantly high mRNA levels of *Cd36* and *Lipa* were observed in mice fed with both HF-HF and MCD groups in comparison with mice fed with a chow diet. Although mice fed with the HF-HF diet also showed increased mRNA expression levels of *Scd-1*, mice in the MCD group did not show a statistically significant outcome (Figure 1E, Supplementary Table S4).

#### 3.1.3. Mice Fed with High HF-HF and MCD Diets Show Distinct Modulation of Metabolic and Biochemical Parameters

**Figure 2:**
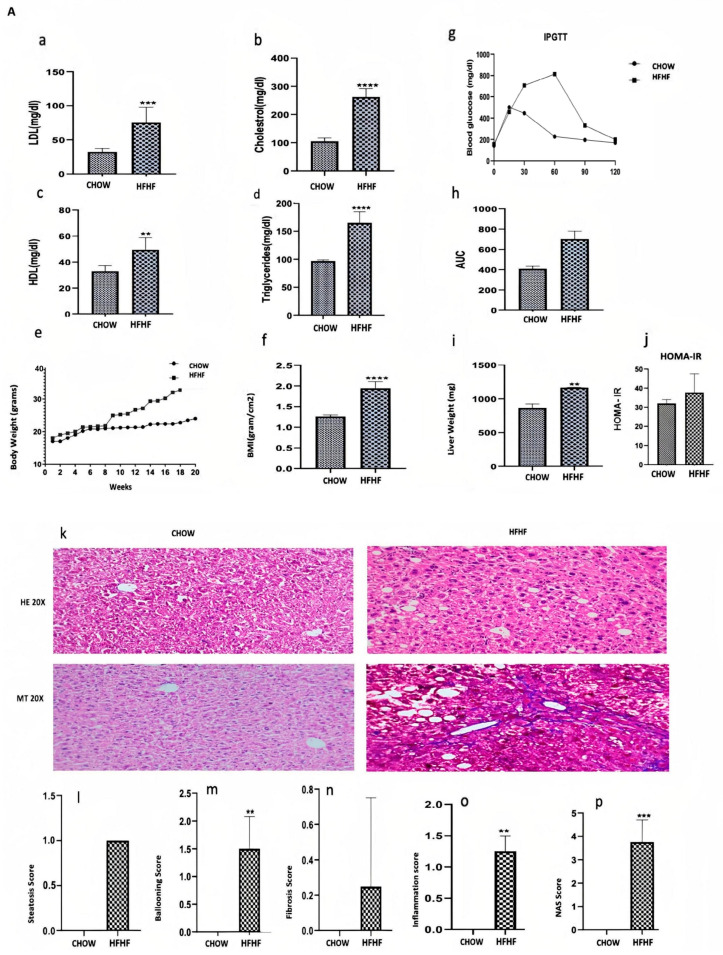

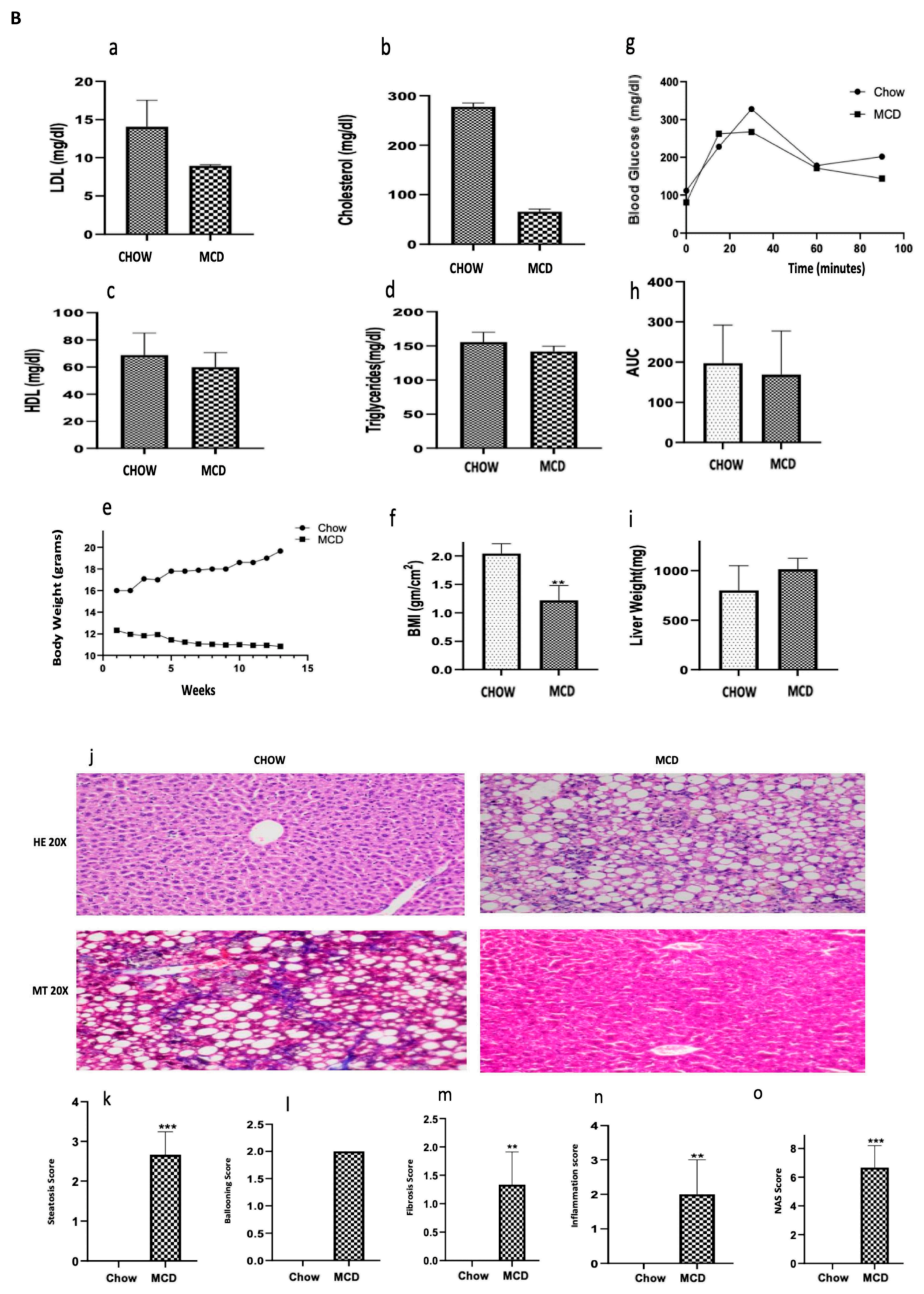
Biochemical, metabolic, and histopathological parameters of chow vs. HF-HF diet and chow vs. MCD diet groups: Lipid profile (**A**) (**a**–**d**) HF-HF group and (**B**) (**a**–**d**) MCD group. Metabolic parameters among the different groups: change in body weight, body mass index (BMI), (**A**) (**e**,**f**) HF-HF group and (**B**) (**e**,**f**) MCD group; intraperitoneal glucose tolerance test (IPGTT) (**A**) (**g**,**h**) HF-HF group and (**B**) (**g**,**h**) MCD group; liver weight (**A**) (**i**) HF-HF group and (**B**) (**i**) MCD group; (**A**) (**j**) increased HOMA-IR in the serum samples of mice in the HF-HF study group. Histopathological analysis showing H&E and MT staining, steatosis, ballooning, and lobular inflammation along with fibrosis and NAS score (**A**) (**k**–**p**) HF-HF group and (**B**) (**j**–**o**) MCD group, respectively. Data are represented as mean ± SEM, *n* = 6 per group, and statistical analysis consisted of one-way ANOVA or two-way ANOVA followed by Bonferroni’s test (** *p* < 0.01; *** *p* < 0.001; **** *p* < 0.0001 for control vs. all other groups).

HF-HF-fed mice exhibited elevated cholesterol, LDL, and triglycerides, reflecting disrupted lipid metabolism along with an increase in body weight and BMI, typical of obesogenic diets (Figure 2A(a,b,d–f), Supplementary Table S4A) as reported by us previously [14,18].

Blood glucose levels in HF-HF-fed mice were also found to be higher compared with those in the chow group. (Figure 2A(g,h), Supplementary Table S4B). HF-HF-fed mice also exhibited a significant increase in the liver weight and a modest non-significant increase in HOMA-IR values in comparison with chow-fed mice (Figure 2A(i,j)), indicating a trend towards greater insulin resistance consistent with known effects of high-fat and sugar diets on fasting glucose and insulin levels [14].

MCD-fed mice on the other hand, showed reduced serum LDL, cholesterol, and triglyceride levels in comparison with chow-fed mice (Figure 2B(a,b,d), Supplementary Table S4A), significant weight loss, lower BMI (Figure 2B(e,f)) with no significant change in blood glucose (Figure 2B(g,h)) as reported previously [13].

#### 3.1.4. Mice Fed with HF-HF and MCD Diets Show Distinct Modulation of Histopathological Parameters

The development of fatty liver disease was analyzed in the HF-HF and MCD groups in terms of semi-quantitative analysis of macrovesicular steatosis, ballooning degeneration, lobular inflammation and fibrosis compared to the chow group. MASH-CRN grading and staging system was used. We observed grossly that the livers of mice in the HF-HF and MCD groups appeared pale, suggesting the deposition of lipid droplets. The mean values of steatosis, ballooning and lobular inflammation along with total score (grade) and stages of mice in both HF-HF (Figure 2A(k–p)) and MCD (Figure 2B(j–o)) study groups were compared.

Overall, both HF-HF and MCD diet-fed mice showed severe pathological parameters compared to chow-fed controls; the MCD group, however, exhibited the highest fibrosis scores.

### 3.2. MASLD Patients Show Significant Hepatic Expression of NLRP3, NLRC4 and AIM2 Along with Their Downstream Signaling Mediators

**Figure 3:**
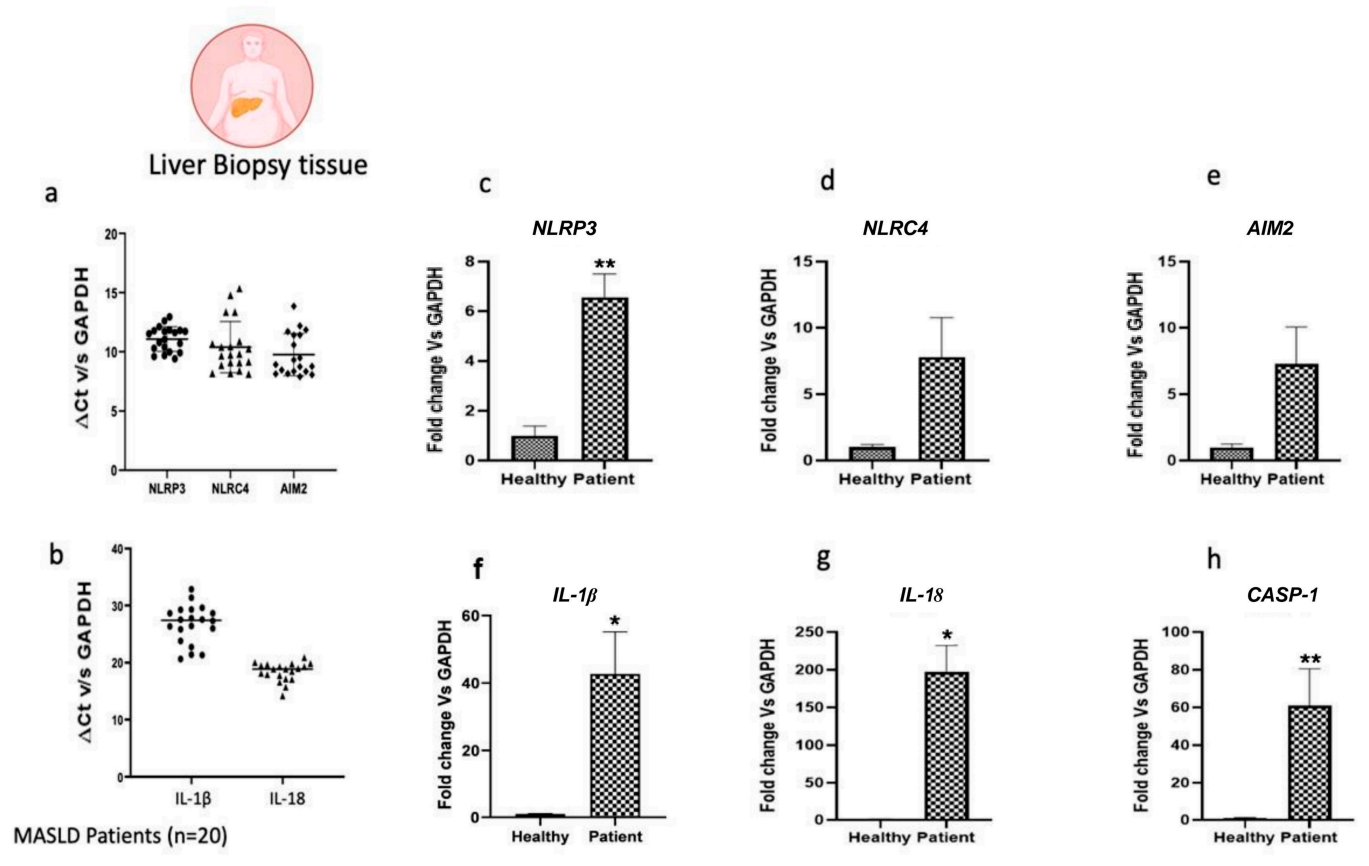
Gene expression profiling of the liver biopsy tissues of MASLD patients, (**a**,**b**) expression levels of *NLRP3*, *NLRC4*, *AIM-2*, *IL-1β* and *IL-18* in MASLD patients (ΔCT values), (**c**–**h**) mRNA expression levels of *NLRP3*, *NLRC4*, *AIM-2*, *IL-1β*, *IL-18*, and *CASPASE-1* in patient samples in comparison with healthy individuals by qRT-PCR. Gene expression is represented as fold change (2^−ΔΔCT^) relative to control and normalized to GAPDH. Data are represented as mean ± SEM, *n* = 6 per group, and statistical analysis consisted of one-way ANOVA or two-way ANOVA followed by Bonferroni’s test (* *p* < 0.05; ** *p* < 0.01 for control vs. all other groups).

As reported previously [19], we found significant levels of *NLRP3* mRNA in the biopsy-proven liver tissue samples from MASLD patients (*n* = 20). Details of the fibro scan data of each patient are provided in Supplementary Table S2. Interestingly, increased mRNA levels of *NLRP3*, *NLRC4* and *AIM2*, along with *IL-1β*, *IL-18* and *CASPASE-1*, were also observed in the liver tissues of these patients, as shown in Figure 3a,b (CT values in patients). We also analyzed the expression of these genes in patient tissue with hepatocytes isolated from healthy individuals, which were obtained from a commercial source (Lonza Group Ltd., Basel, Switzerland) (Figure 3c–h). Our data demonstrate significantly increased mRNA expression of *NLRP3* in patient samples compared with healthy controls. However, although *NLRC4* and *AIM2* exhibited a trend toward higher expression in patients, these differences did not reach statistical significance. Further analysis with a larger sample size is therefore warranted to validate these observations.

### 3.3. In Vitro Experiments

#### 3.3.1. In Vitro Treatment of HepG2 Cells with Free Fatty Acids Shows Increased Expression of IL-1β and IL-18

**Figure 4:**
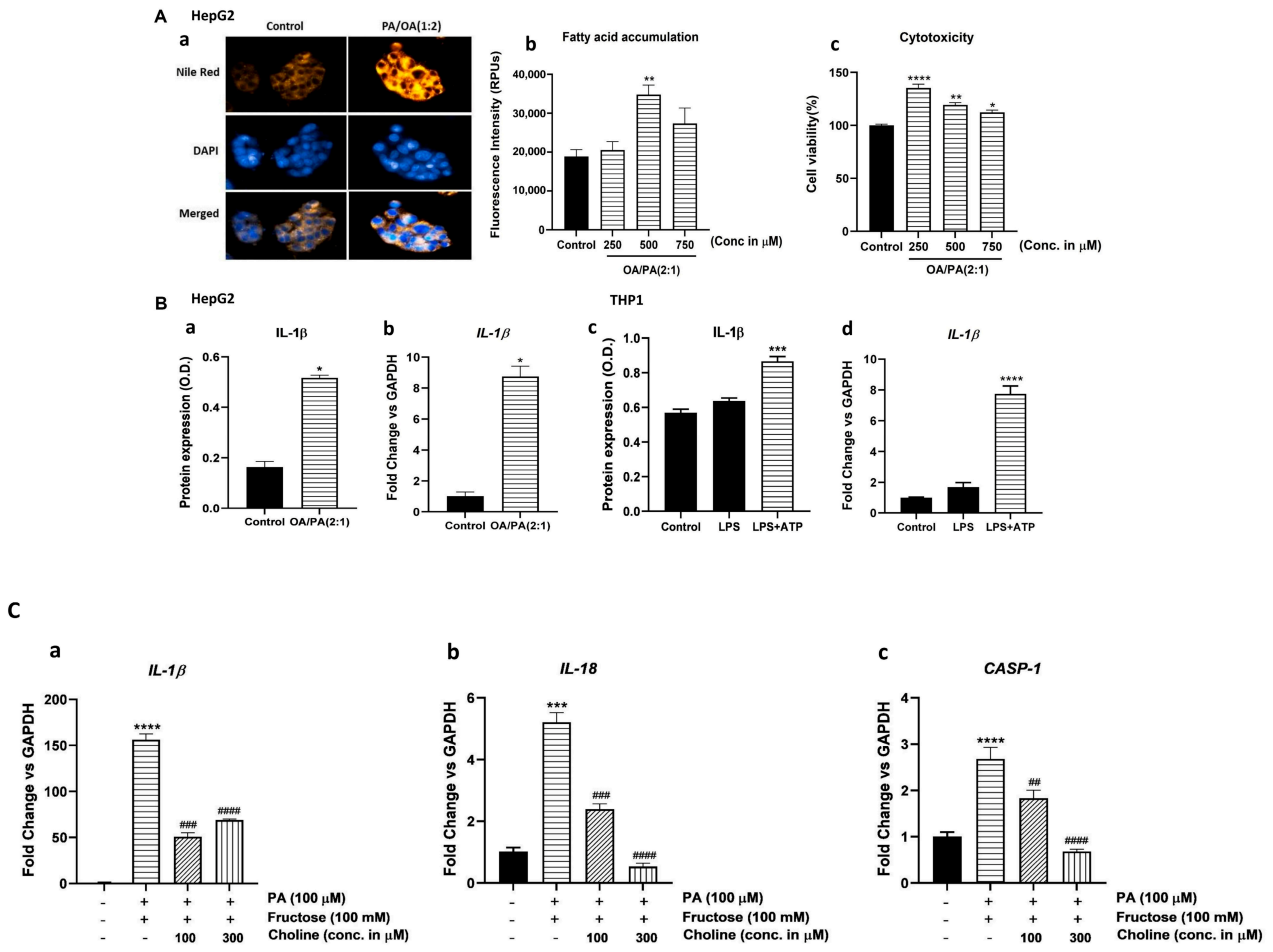
In vitro experiments suggesting the activation of the inflammasome pathway in MASLD, (**A**) (**a**,**b**) induction of fatty acid accumulation in HepG2 cells as a result of challenge with fatty acids (2:1 mixture of oleic acid and palmitic acid using Nile Red and DAPI staining by microscopic and fluorescence readout methods); (**A**) (**c**) effect of varying concentrations of fatty acids on cellular viability in HepG2 cells by MTT method; (**B**) (**a**,**b**) fatty acid-induced increase in the protein and mRNA levels of *IL-1β* in HepG2 cells, respectively; (**B**) (**c**,**d**) LPS+ATP-induced increase in the levels of protein and mRNA levels of *IL-1β* in THP-1 cells, respectively, (**C**) (**a**–**c**) effect of choline on palmitic acid (PA)+fructose-induced inflammation in a co-culture model using THP-1 and HepG2 cells as analyzed by the mRNA levels of *IL-1β*, *IL-18*, and *CASPASE-1*. Gene expression is expressed as fold change (2^−ΔΔCT^) relative to control and normalized to *GAPDH*. Data are represented as mean ± SEM, *n* = 6 per group, and statistical analysis consisted of one-way ANOVA or two-way ANOVA followed by Bonferroni’s test (* *p* < 0.05; ** *p* < 0.01; *** *p* < 0.001; **** *p* < 0.0001 for control vs. all other groups and ## *p* < 0.01; ### *p* <0.001; #### *p* < 0.0001, compared to treatment by the combination of palmitic acid and fructose vs. all other groups).

We further validated inflammasome activation using in vitro models of metabolic dysfunction-associated fatty liver disease, where we observed increased expression of IL-1β at both mRNA and protein levels. HepG2 cells treated with a 2:1 mixture of oleic acid and palmitic acid (OA+PA) showed fatty acid accumulation as shown by an increase in fluorescence after Nile Red staining without any apparent toxicity up to 750 µM as analyzed by MTT assay (Figure 4A(a–c)).

Treatment of OA+PA also showed an increase in the levels of IL-1β in HepG2 cells, both at the levels of protein and mRNA (Figure 4B(a,b)).

#### 3.3.2. In Vitro Treatment of Differentiated THP-1 Cells with LPS and ATP Shows Increased Expression of IL1β and IL-18

Treatment of differentiated THP-1 cells with LPS+ATP showed activation of the inflammasome, as shown by the increase in the protein and mRNA levels of IL-1β (Figure 4B(c,d)). Priming of the differentiated THP-1 cells with LPS (100 ng/mL, 2 h) led to transcription of inflammasome-related genes such as pro-IL-1β and IL-18. This was followed by stimulation with ATP (2.5 mM, 2 h), as the second signal required for inflammasome activation.

#### 3.3.3. Treatment of the HepG2/THP-1 Cells with Choline Shows Inhibition of Palmitic Acid and Fructose-Induced Increase in the Levels of IL-1β, IL-18 and CASPASE-1

**Scheme 4:**
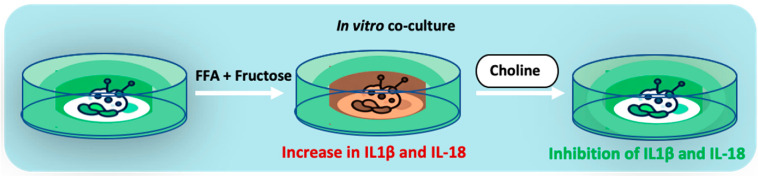
Summary of findings from in vitro co-culture experiment.

A co-culture of THP-1 and HepG2 cells was established. Differentiated THP-1 cells were mixed with HepG2 cells in a ratio of 1:5. The THP-1 and HepG2 co-culture was pretreated with choline at 100 µM and 300 µM for 30 min, followed by treatment with a combination of palmitic acid (100 µM) and fructose (100 mM) for 48 h. We observed increased mRNA expression of *IL-1β*, *IL-18* and *CASPASE-1* in co-culture conditions, and supplementation of choline at 100 µM and 300 µM led to inhibition of palmitic acid and fructose-induced increase in the mRNA levels of *IL-1β*, *IL-18* and *CASPASE-1* (Figure 4C(a–c)). Summary of findings from in vitro co-culture experiment is shown in Scheme 4.

## 4. Discussion

Mice fed a high-fat, high-fructose (HF-HF) diet showed altered metabolic parameters such as obesity, insulin resistance, and fatty liver over 20 weeks. However, they did not develop MASH. In contrast, the methionine- and choline-deficient (MCD) diet, although it showed reduced levels of plasma triglycerides and body weight, led to accumulation of fat and development of fibrosis in the liver tissue within 10 weeks, mimicking the pathology of human MASH. These findings align with previous reports that suggest the importance of methionine and choline in lipid metabolism and VLDL-mediated triglyceride export [15,16,17,19,20,21].

Previous reports suggest that dysregulated inflammasomes play an important role in the progression of MASLD. However, very few studies have systematically compared the key members of the canonical inflammasome family in a unified setup. A comparative analysis of the relative expression patterns of these inflammasomes in our study showed significantly elevated mRNA levels of *NLRP3*, *NLRC4*, *AIM2*, *IL-1β*, and *IL-18* in liver biopsies from MASLD patients, which were further validated in mouse models. Our findings are consistent with several clinical and experimental studies demonstrating upregulated hepatic expression of NLRP3 inflammasome components and downstream cytokines in patients with MASLD/MASH, indicating a pivotal role of innate immune activation in disease progression [8,9,10,11,12]. However, data on the role of NLRC4 and AIM2 inflammasomes in MASLD are scarce in comparison with NLRP3, emphasizing the relevance of this present comparative analysis [12].

We observed elevated levels of *Nlrp3*, *Il-1β*, and *Il-18* in the liver tissues of mice fed with both HF-HF and MCD diets. However, expression levels of *Nlrc4* and *Aim2* were selectively induced in the mice fed with the MCD diet, indicating diet-specific activation of inflammasomes. The MCD diet also resulted in a more severe cytokine response compared with the HF-HF diet, which also correlated well with stronger hepatic inflammation and fibrosis by histological analysis, as shown by higher NAS and fibrosis scores in MCD-fed mice compared to HF-HF and chow groups. However, direct comparison of the HF-HF and MCD models should be interpreted with caution as the two dietary interventions differ significantly in nutritional composition, duration of feeding, body weight changes, plasma lipid profile and overall disease phenotype. The more severe inflammatory response and increased expression of inflammasome-related genes in the MCD model may thus be indicative, at least in part, of increased liver injury and fibrosis in this model. However, the data suggest that inflammasome-related pathways are differentially regulated in different dietary models of MASLD. This observation is consistent with previous studies showing that the magnitude of inflammasome activation correlates closely with the severity of disease, hepatocellular injury and fibrosis, but not with steatosis alone [10,11,12,13,18].

While NLRP3 inflammasome activation in MASLD is well established, the mechanisms underlying NLRC4 and AIM2 activation remain less defined. The differential activation of inflammasomes between dietary models could possibly be due to fundamental differences in the severity and nature of liver injury. Increasing evidence suggests the role of AIM2 as a key guardian of cellular integrity. The pronounced hepatocellular injury and oxidative stress induced by the MCD diet likely promote the release of damage-associated molecular patterns, including mitochondrial and nuclear DNA, which may directly activate the AIM2 inflammasome through cytosolic DNA sensing that activates Caspase-1, leading to IL-1β and IL-18 secretion and pyroptotic cell death [22,23]. Recent studies have similarly proposed that mitochondrial dysfunction, oxidative stress and DNA release serve as important triggers for AIM2 inflammasome activation in inflammatory disorders [22,23].

In parallel, increased gut permeability and microbial translocation in MCD-fed mice may provide pathogen-associated molecular patterns capable of engaging NLRC4 in hepatic immune cells. In an MCD diet-induced mouse model, intestinal permeability changes occurred following early liver injury and TNFα induction, supporting a link between hepatic injury and disrupted gut barrier function in MASH [24,25]. This is supported by growing evidence highlighting the role of the gut-liver axis in MASLD progression, whereby increased intestinal permeability and microbial products contribute to hepatic inflammation and innate immune activation [19,24,25].

HF-HF feeding, on the other hand, although it induces steatosis, creates low-grade metabolic inflammation, which may be sufficient to activate NLRP3 but causes limited hepatocyte death, mitochondrial disruption, and gut barrier dysfunction, which may not generate sufficient activation signals for AIM2 (cytosolic DNA) and NLRC4 (microbial components). In a study reported by Machado et al. (2025) [26], a Western diet (45% energy from fat, predominantly saturated fat, with 0.2% cholesterol, and drinking water supplemented with fructose and glucose for 16 weeks) more closely mimics human MASH compared with the MCD diet but induces milder and less consistent liver injury. In contrast, the MCD diet more robustly reproduces the pathogenic mechanisms driving progression to advanced MASH, including hepatocyte death, inflammation, fibrosis, and cellular stress pathways. Together, these mechanisms may explain the broader inflammasome activation observed in the MCD model.

We also observed increased expression of NLRP3 mRNA in the biopsy-proven liver tissue samples from MASLD patients (*n* = 20) in comparison with commercially obtained primary human hepatocytes, which corroborates our findings from mouse models, as reported previously [19]. The cohort includes patients with a spectrum of disease severity ranging from simple steatosis to definite NASH and fibrosis stages F0–F3, thereby allowing assessment across different stages of MASLD progression. However, the unavailability of liver tissue/biopsy samples from healthy humans was a limitation of this study. Notably, our observations are consistent with previous studies demonstrating increased inflammasome activation in patients with steatohepatitis and advanced liver disease [8,10,11,12].

To validate these findings, we used in vitro models. HepG2 cells treated with free fatty acids and THP-1 macrophages stimulated with LPS+ATP showed increased *IL-1β* at both mRNA and protein levels, confirming inflammasome activation. Our previous organoid-based studies further support this: liver organoids derived from healthy hepatocytes after palmitic acid exposure or those developed from MASLD patient tissues showed elevated *IL-1β*, *IL-18*, and *CASPASE-1*, mimicking in vivo inflammation [27]. In a co-culture experiment using HepG2 and differentiated THP-1 cells, on the other hand, supplementation of choline at 100 µM and 300 µM led to inhibition of palmitic acid- and fructose-induced increases in the mRNA levels of *IL-1β*, *IL-18*, and *CASPASE-1*.

Findings reported in this manuscript suggest that diet-induced inflammasome activation is a key driver in the progression of MASLD. These findings also indicate that choline serves as a significant dietary regulator of inflammasome activation in MASLD. Choline deprivation worsens hepatic inflammation caused by inflammasomes in mice, and supplementation of choline in an in vitro system mitigates the cytokine response triggered by metabolic stress. Adding choline to the diet, therefore, may help treat MASLD by affecting the inflammasome axis. We also recommend the use of various in vitro and in vivo models used in our study as valuable tools for the identification of therapeutics targeting the inflammasome axis and for understanding the pathophysiology of MASLD.

## 5. Conclusions

Findings reported in this manuscript demonstrate that the choline deficiency using the MCD diet elicits significantly stronger immune activation, inflammatory cytokine release, and hepatic injury than the HF-HF diet, highlighting its importance in MASLD progression. Supplementation with dietary choline in an in vitro setup, on the other hand, mitigates the metabolic stress-induced inflammation.

Given the similarity between the MCD mouse model and MASH in lean individuals with mild metabolic dysfunction but advanced fibrosis, we hereby recommend examining the expression of canonical inflammasomes in non-obese MASLD patients in the future. Future work with larger patient cohorts, detailed dietary assessments, and physiologically relevant humanized models will further help and strengthen the translational relevance of these findings.

## Figures and Tables

**Table 1:** Primer sequences used in the study for gene expression analysis.

Gene	Forward Primer	Reverse Primer
Mouse Primers		
*Nlrp3*	5′-TCACAACTCGCCCAAGGAGGAA-3′	3′-AAGAGACCACGGCAGAAGCTAG-5′
*Il-18*	5′-GACAGCCTGTGTTCGAGGATATG-3′	3′-TGTTCTTACAGGAGAGGGTAGAC-5′
*Il-1* *β*	5′-TGGACCTTCCAGGATGAGGACA-3′	3′-GTTCATCTCGGAGCCTGTAGTG-5′
*Casp1*	5′-GGCACATTTCCAGGACTGACTG-3′	3′-GCAAGACGTGTACGAGTGGTTG-5′
*Aim2*	5′-AGGCTGCTACAGAAGTCTGTCC-3′	3′-TCAGCACCGTGACAACAAGTGG-5′
*Nlrc4*	5′-CTCACCACGGATGACGAACAGT-3′	3′-TGTCATCCAGTATGAGTCTCTCG-5′
*Ace2*	5′-TCCATTGGTCTTCTGCCATCCG-3′	3′-AGACCATCCACCTCCACTTCTC-5′
*Cxcl1*	5′-TCCAGAGCTTGAAGGTGTTGCC-3′	3′-AACCAAGGGAGCTTCAGGGTCA-5′
*Il6*	5′-TACCACTTCACAAGTCGGAGGC-3′	3′-CTGCAAGTGCATCATCGTTGTTC-5′
*Tnf-* *α*	5′-CAGGCTCTGGAGAACAGCACAT-3′	3′-GACACATGCTGGCTTCTCTTCC-5′
*Cd36*	5′-GGACATTGAGATTCTTTTCCTCTG-3′	3′-GCAAAGGCATTGGCTGGAAGAAC-5′
*Fasn*	5′-CACAGTGCTCAAAGGACATGCC-3′	3′-CACCAGGTGTAGTGCCTTCCTC-5′
*Lipa*	5′-ATCCTGGTGAGGAACACTCGGT-3′	3′-TAGAATCTGCCAGCAAGCCGTG-5′
*Scd1*	5′-GCAAGCTCTACACCTGCCTCTT-3′	3′-CGTGCCTTGTAAGTTCTGTGGC-5′
*α* *-sma*	5′-TGCTGACAGAGGCACCACTGAA-3′	3′-CAGTTGTACGTCCAGAGGCATAG-5′
*Col1a1*	5′-CCTCAGGGTATTGCTGGACAAC-3′	3′-CAGAAGGACCTTGTTTGCCAGG-5′
*Tgf-* *β*	5′-TGATACGCCTGAGTGGCTGTCT-3′	3′-CACAAGAGCAGTGAGCGCTGAA-5′
*Asc/pycad*	5′-CTGCTCAGAGTACAGCCAGAAC-3′	3′-CTGTCCTTCAGTCAGCACACTG-5′
*Gasd*	5′-GGTGCTTGACTCTGGAGAACTG-3′	3′-GCTGCTTTGACAGCACCGTTGT-5′
*Cd33*	5′-GCATCTGATGCTGTGACTCCAG-3′	3′-AGTGTGGACACTGCTCTGTTCC-5′
*Gapdh*	5′-CATCACTGCCACCCAGAAGACTG-3′	3′-ATGCCAGTGAGCTTCCCGTTCAG-5′
Human Primers		
*NLRP3*	5′-GGACTGAAGCACCTGTTGTGCA-3′	3′-TCCTGAGTCTCCCAAGGCATTC-5′
*NLRC4*	5′-AGGTCCCACAACTCGTCAAGCT-3′	3′-TGCTCACACGATTTCCCGCCAA-5′
*AIM2*	5′-GCTGCACCAAAAGTCTCTCCTC-3′	3′-CTGCTTGCCTTCTTGGGTCTCA-5′
*IL-1* *β*	5′-CCACAGACCTTCCAGGAGAATG-3′	3′-GTGCAGTTCAGTGATCGTACAGG-5′
*IL-18*	5′-GATAGCCAGCCTAGAGGTATGG-3′	3′-CCTTGATGTTATCAGGAGGATTCA-5′
*GAPDH*	5′-GTCTCCTCTGACTTCAACAGCG-3′	3′-ACCACCCTGTTGCTGTAGCCAA-5′

## Data Availability

The original contributions presented in the study are included in the article and in the online Supplementary Material. Further inquiries can be directed to the corresponding author.

## Supplementary Materials

The following supporting information can be downloaded at: https://www.mdpi.com/article/10.3390/cells15141298/s1, Table S1: Composition of diets used in this study; Table S2: Clinical anthropometric data of NAFLD patients; Table S3: Differential Expression of genes in MCD and HF-HF Diet; Table S4: Lipid profile, liver weight and IPGTT profile of mice fed with HF-HF and MCD diets.

## Abbreviations

MASLD	Metabolic dysfunction-associated steatotic liver disease
NLRP3	Nucleotide-binding oligomerization domain (NOD), leucine-rich repeat (LRR) (NLRs) containing protein 3
NLRC4	NLR family CARD domain-containing protein 4
AIM2	Absent in melanoma 2
α-SMA	alpha-smooth muscle actin
Col1a1	Collagen type 1 α1
TGF-β	Transforming growth factor beta
FASN	Fatty acid synthase
SCD-1	Stearoyl-CoA Desaturase-1
CD36	Cluster of differentiation 36 or fatty acid translocase (FAT)
