# Supplementary material for: An Integrated Analysis to Understand the Dysregulation of Innate Immune Response in Mouse Models of MASLD
Source: Cells. 2026 Jul 21;15(14):1298. doi: 10.3390/cells15141298 (PMC13407229; doi:10.3390/cells15141298)
Supplement: Supplementary file 1 [file cells-15-01298-s001.zip › cells-4383569-supplementary.pdf]

# Supplementary Material

| | | | | |
|---|---|---|---|---|
| **Table S1**<br>**Composition of different diets used in the study** | | | | |
| **Class** | **Ingredients** | | | |
| | | **Chow** | **MCD** | **HFHF** |
| Diet company & code | | **1324P (Altromin international, Germany)** | **A02082002BR (Research Diet Inc., USA)** | **D16030909 (Research Diet Inc., USA)** |
| Protein | Casein, Lactic, 30 Mesh | | | 200.00g |
| Protein | Glutamine, L | | 40.00g | |
| Protein | Glycine | | 23.30 g | |
| Protein | Lysine, L, HCl | | 18.00 g | |
| Protein | Arginine, L | 11,175 mg | 12.10 g | |
| Protein | Leucine, L | | 11.10 g | |
| Protein | Threonine, L | | 8.20 g | |
| Protein | Isoleucine, L | | 8.20 g | |
| Protein | Valine, L | | 8.20 g | |
| Protein | Phenylalanine, L | | 7.50 g | |
| Protein | Asparagine, L, Monohydrate | | 6.00 g | |
| Protein | Tyrosine, L | | 5.00 g | |
| Protein | Histidine, L, HCl, H2O | | 4.50 g | |
| Protein | Alanine, L | 8,292mg | 3.50 g | |
| Protein | Aspartic Acid, L | 15,297mg | 3.50 g | |
| Protein | Cystine, L | 3,182mg | 3.50 g | 3.00 g |
| Protein | Proline, L | 12,523mg | 3.50 g | |
| Protein | Serine, L | 8,965mg | 3.50 g | |
| Protein | Methionine, L | 2,709mg | - | |
| Protein | Tryptophan, L | | 1.80 g | |
| Carbohydrate | Sucrose, Fine Granulated | | 455.30 g | 4.00 g |
| Carbohydrate | Starch, Corn | | 150.00 g | |
| Carbohydrate | Lodex 10 | | 50.00 g | |
| | Maltodextrin 10 | | | |
| Carbohydrate | Fructose | | | 193.80 g |

| | | | | |
|---|---|---|---|---|
| Fiber | Solka Floc, FCC200 | | 30.00 g | 50.00 g |
| | Cellulose, BW200 | | | |
| Fat | Corn Oil | | 100.00 g | |
| Fat | Soybean Oil, USP | | | 25.00 g |
| Fat | Lard | | | 245.00 g |
| Mineral | S10001 | | 35.00 g | |
| Mineral | S10026 | | | 50.00 g (S10026 B) |
| Mineral | Sodium Bicarbonate | | 7.50 g | |
| Vitamin | V10001 | | 10.00 g | 1.00 g (V10001 C) |
| Vitamin | Choline Bitartrate | | - | 2.00 g |
| Vitamin | Choline chloride | 600mg | | |
| DiCalcium Phosphate | | | | |
| Calcium Carbonate | | | | |
| Potassium Citrate, 1 H2O | | | | |
| Dye | Dye, Blue FD&C #1, Alum. Lake 35-42% | | | 0.03 g |
| Dye | FD&C Blue Dye #1 | | | |
| Dye | Dye, Yellow FD&C #5, Alum. Lake 35-42% | | 0.05 g | 0.03 g |

# Table S2

# Clinical and anthropometric data of NAFLD patients

| S.N. | Date | Patient Details | | | Biopsy Report | | | | | | | | | | | | | | | | |
|---|---|---|---|---|---|---|---|---|---|---|---|---|---|---|---|---|---|---|---|---|---|
| | | Patient ID | Gender | Age | NAS score | NASH | Stage | Fibroscan date | LSM | IQR | CAP | IQR | CMRF TG | CMRF-HDL | CMRF-FBS | CMRF-HTN | CMRF-Obesity | CMRF Total | Metabolic syndrome | Medicine | NAFLD activity score | NAS |
| 1 | 20-06-2022 | 105447918 | M | 40 | 5 | 1 | 1a | 20-Apr-22 | 5 | 0.3 | 319 | 40 | | | | | | | | | 0-3 | Steatosis |
| 2 | 29-06-2022 | 105949581 | F | 56 | 5 | 1 | 3 | 16-Jun-22 | 6 | 0.6 | 320 | 17 | 1 | 1 | 1 | 0 | 1 | 4 | 1 | | 0-3 | Lobular inflammation |
| 3 | 04-07-2022 | 105447833 | M | 30 | | | | | | | | | | | | | | | | | 0-2 | hepatocyte ballooning |
| 4 | 07-07-2022 | 106026145 | f | 60 | 3 | 1 | 4 | 05-Jul-22 | 10.2 | 1.6 | 350 | 39 | 0 | 0 | 1 | 0 | 1 | 2 | 0 | | Total = 8 | > 5 definite NASH |
| 5 | 13-07-22 | 102497659 | F | 56 | 0 | 0 | 0 | 12-Nov-22 | 8.7 | 0.9 | 227 | 39 | 0 | 0 | 1 | 1 | 1 | 3 | 1 | Glycomet, Amlodipine | | <3 Not NASH |
| 6 | 14-07-2022 | 105979642 | M | 21 | 4 | 1 | 0 | 05-Apr-22 | 6.8 | 0.2 | 233 | 16 | 0 | 0 | 0 | 0 | 1 | 1 | 0 | | | |
| 7 | 03-08-2022 | 104153094 | M | 52 | 4 | 1 | 3 | 12-Jul-22 | 7.6 | 1.4 | 359 | 31 | 0 | 0 | 1 | 0 | 1 | 2 | 0 | Metformin SR 500 mg | | |
| 8 | 03-08-2022 | 104895254 | F | 42 | 3 | 0 | 1a | 27-Oct-22 | 6.8 | 0.8 | 356 | 39 | 0 | 0 | 1 | 0 | 1 | 2 | 0 | Metformin SR 500 mg | | |
| 9 | 24-08-2022 | 106078957 | M | 69 | 3 | 1 | 2 | 04-Jul-22 | 18.7 | 3.3 | 378 | 13 | 0 | 0 | 1 | 1 | 1 | 3 | 1 | Cilnidipine | | |
| 10 | 24-08-2022 | 105998601 | F | 35 | 3 | 1 | 1a | 04-Jun-22 | 7.8 | 0.5 | 293 | 18 | 0 | 1 | 0 | 0 | 1 | 2 | 0 | | | |
| 11 | 09-09-2022 | 106072911 | F | 39 | 7 | 1 | 2 | 17-Jul-22 | 19 | 2 | 336 | 26 | | | | | | | | | | |
| 12 | 09-09-2022 | 105970061 | M | 38 | 3 | 1 | 1c | 02-Jun-22 | 7.8 | 1.3 | 270 | 19 | 0 | 0 | 0 | 0 | Ra | 0 | 0 | | | |
| 13 | 19-11-2022 | 104824037 | M | 55 | 4 | 1 | 2 | 05-Jul-22 | 12.3 | 1.7 | 383 | 37 | | | | | | | | Saroglitazar, Inj. Insulin | | |
| 14 | 15-09-2022 | 106117618 | F | 20 | 5 | 1 | 1a | 30-Jul-22 | 6.4 | 1.2 | 287 | 21 | 1 | 1 | 0 | 0 | 1 | 3 | 1 | | | |
| 15 | | 106235934 | M | 28 | 3 | 1 | 3 | 15-Sep-22 | 12.2 | 2.5 | 230 | 17 | | | | | | | | | | |
| 16 | 07-11-2022 | 105781333 | M | 31 | 0 | 0 | 0 | 28-Jul-22 | 6.1 | 0.6 | 198 | 14 | | | | | | | | | | |
| 17 | 15-08-2022 | 106117618 | M | 26 | 4 | 1 | 1a | | | | | | | | | | | | | | | |
| 18 | 13-09-2022 | 101399518 | M | 40 | 5 | 1 | 1b | 13-Aug-22 | 7.8 | 1.7 | 277 | 9 | | | | | | | | | | |
| 19 | | 106200192 | F | 29 | | | | 24-Aug-22 | 14.5 | 6 | 232 | 5 | | | | | | | | | | |
| 20 | 04-11-2022 | 105535634 | M | 48 | 5 | 1 | 3 | 17-Oct-22 | 13.2 | | 323 | | 0 | 1 | 0 | 0 | 1 | 2 | 0 | | | |
| 21 | | 102549364 | F | 48 | 4 | 1 | 2 | 18-Aug-22 | 5.1 | 1 | 397 | 8 | 1 | 1 | 1 | 0 | 1 | 4 | 1 | Metformin SR 500 mg | | |
| 22 | | 103291516 | M | 26 | 6 | 1 | 0 | 02-Jun-22 | 6.1 | 0.7 | 351 | 15 | 0 | 1 | 0 | 0 | 1 | 2 | 0 | | | |

LSM-Liver Stiffnesement; IQR-Interquartile Range; CAP- Controlled Attenuation Parameter; CMRF-Cardio-Metabolic Risk Factor; TG-Triglyc-High Density Lipoproteins; HTN-Hypertension; FBS-Fasting Blood Sugar

# Table S3

## Differential Expression of Genes in MCD and HF-HF Diet

| Gene Expression | MCD diet | HF-HF diet | Relevance |
|---|---|---|---|
| NLRP3 | | | |
| ASC | | | |
| IL-1β | | | |
| IL-18 | | | |
| CASP1 | | | |
| GASD | | | Inflammation |
| AIM2 | | | |
| NLRC4 | | | |
| CXCL1 | | | |
| IL-6 | | | |
| TNF-α | | | |
| CD36 | | | Fatty Acid Uptake & Lipogenesis |
| LIPA | | | |
| FASN | | | |
| SCD1 | | | |
| COL1A1 | | | |
| α-SMA | | | Fibrosis |
| TGF-β | | | |

**Table S4**

**Lipid profile, liver weight and IPGTT profile of mice fed with HF-HF and MCD diets**

**4A: Lipid Profile and liver weight of mice fed with HF-HF and MCD diets**

| Study | Group | Cholesterol(mg/dL) | LDL (mg/dL) | HDL (mg/dL) | Triglyceride(mg/dL) | Liver Weight(mg) |
|---|---|---|---|---|---|---|
| HFHF Study | Chow | 105.20±10.89 | 32.50±5.42 | 33.08±3.89 | 96.93±1.98 | 862.50±122.85 |
| | HFHF | 262.79±28.90 | 77.41±21.07 | 49.93±8.41 | 165.69±20.37 | 1162.00±12.12 |
| MCD Study | Chow | 117.67±8.25 | 14.07±3.45 | 68.85±16.31 | 155.41±14.70 | 801.16±248.01 |
| | MCD | 167.28±16.97 | 8.92±0.16 | 59.85±10.82 | 141.78±7.73 | 1013.13±111.69 |

**4B: IPGTT profile of mice fed with HF-HF and MCD diets**

| Time (Min) | Glucose concentration (mg/dL) | | | | | |
|---|---|---|---|---|---|---|
| | HFHF Study | | | MCD Study | | |
| | Chow (mg/dL) | HFHF | | | MCD | |
| | | mg/dL | % Change from 0 min | Chow (mg/dL) | mg/dL | % Change from 0 min |
| 0 | 143.00±17.22 | 155.00±22.05 | 100 | 111.33±25.50 | 80.67±9.45 | 100 |
| 15 | 501.40±418.30 | 457.33±84.68 | 295 | 228.00±118.16 | 262.67±109.97 | 327 |
| 30 | 445.00±428.39 | 706.67±384.59 | 455 | 327.67±72.06 | 267.33±30.92 | 334 |
| 60 | 227.40±72.96 | 814.33±424.15 | 525 | 177.67±36.56 | 170.67±24.01 | 211 |
| 90 | 195.60±18.68 | 330.67±112.35 | 169 | 201.67±25.32 | 143.67±48.58 | 179 |
| 120 | 168.40±11.59 | 199.17±33.17 | 128 | - | - | - |
